# Facultative CAM and photosynthetic electron transport: unravelling the salinity acclimation puzzle in *Mesembryanthemum crystallinum*

**DOI:** 10.3389/fpls.2026.1753517

**Published:** 2026-01-29

**Authors:** Maria Pilarska

**Affiliations:** The Franciszek Górski Institute of Plant Physiology, Polish Academy of Sciences, Kraków, Poland

**Keywords:** crassulacean acid metabolism, halophyte, ice plant, photosynthetic light reactions, plastoquinone pool redox state, soil salinity

## Abstract

Progressive soil salinisation is a major constraint to agriculture, and deciphering resistance strategies in plants adapted to such harsh environments has the potential for improving salinity tolerance in crops. Efficient regulation of the photosynthetic light reactions is a key element of acclimation to adverse environmental conditions, as it ensures optimal production of reducing power and ATP. The annual halophyte *Mesembryanthemum crystallinum* has long served as a model plant for studying stress response mechanisms. In this species, exposure to salt stress induces a transition from C_3_ photosynthesis to crassulacean acid metabolism (CAM). This review summarises the latest findings on the regulation of photosynthetic electron transport (PET) under salinity in *M. crystallinum*, focusing on their potential dependence on CAM. It also suggests a model in which the plastoquinone pool plays a major role in PET acclimation.

## 1 Introduction

Soil salinisation results from natural factors, like weathering of rocks, seawater submergence, high evaporation and low precipitation in hot climates, but also from human activities such as inappropriate irrigation practices and excessive fertilisation (Tarolli et al., 2024). For plants, high salt concentration is considered a major environmental threat, often leading to ion imbalance, hyperosmotic stress, oxidative damage, and nutrient deficiency, which can restrict the growth and development of crops and natural ecosystems (Shahid et al., 2020). Saline environments are expected to expand in the coming decades due to global climate change causing the rise of sea levels and intensified irrigation of crops. Therefore, identifying the mechanisms underlying salinity tolerance in plants remains an important challenge.

**Figure 1:**
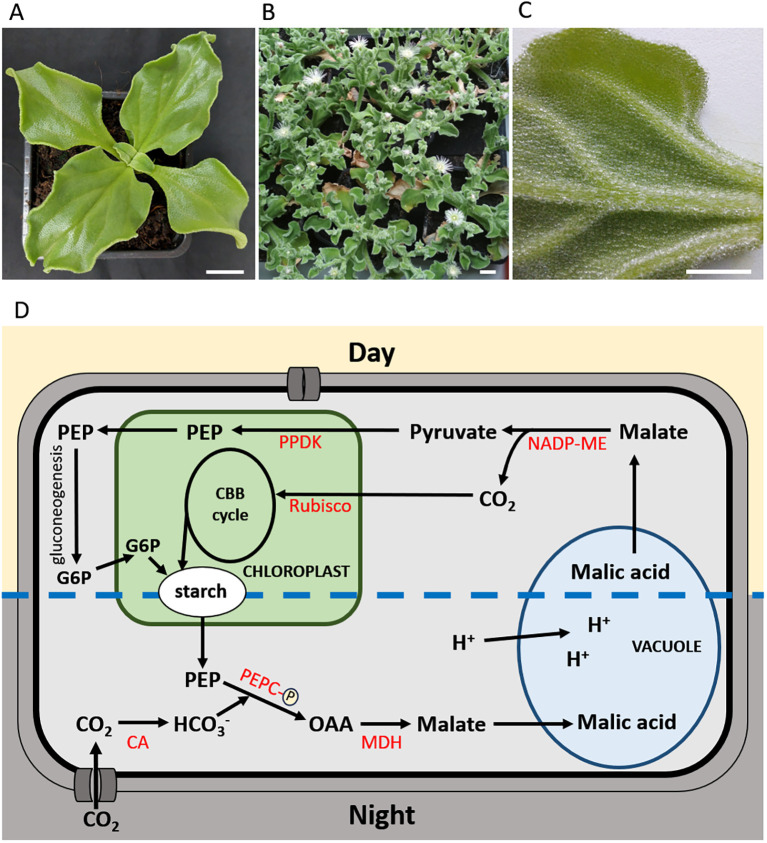
**(A-C)***Mesembryanthemum crystallinum* plants: **(A)** juvenile stage, **(B)** flowering stage, **(C)** leaf abaxial surface with visible bladder cells. **(A, B)** Bar = 2 cm; **(C)** Bar = 1 cm; **(D)** Simplified diagram of major CAM metabolite fluxes in *M. crystallinum*. This model is based on information summarised in Holtum et al. (2005) and Borland et al. (2009). CA, carbonic anhydrase; CBB, Calvin-Benson-Bassham; G6P, glucose-6-phosphate; MDH, malate dehydrogenase; NADP-ME, NADP-dependent malic enzyme; OAA, oxaloacetate; PEP, phosphoenolpyruvate; PEPC, phosphoenolpyruvate carboxylase; PPDK, pyruvate orthophosphate dikinase; Rubisco, ribulose-1,5-bisphosphate carboxylase/oxygenase.

Among halophytes that can grow and reproduce in saline conditions is the model plant, annual *Mesembryanthemum crystallinum* L. (common ice plant), belonging to the Aizoaceae family. Native to southern and eastern Africa, *M. crystallinum* is now widely distributed in regions with a Mediterranean climate (Adams et al., 1998). The ice plant is well known for the induction of crassulacean acid metabolism (CAM) in response to high salinity and for its distinct large epidermal bladder cells, where salt is sequestered (Winter and Lüttge, 1976; Adams et al., 1998) (**Figures 1A-C**). The development of nocturnal CO_2_ fixation represents an extreme acclimation strategy to saline conditions, which has made this halophyte one of the most extensively studied model plants for investigating mechanisms underlying salinity tolerance (Thomas and Bohnert, 1993; Kore-Eda et al., 2004; Libik et al., 2005; Li et al., 2021). However, results obtained with this species are handicapped by a persistent problem: which effects are caused solely by salinity and which are dependent on CAM? One of the crucial elements of stress acclimation is the maintenance of efficient photosynthetic electron transport (PET), which ensures sufficient ATP and NADPH production while limiting the formation of reactive oxygen species (ROS). As induction of CAM changes the metabolic and energetic fluxes in chloroplasts, it can be expected that it also affects photosynthetic light reactions. Based on recent studies, this mini review outlines the mechanisms of PET acclimation to salinity in *M. crystallinum* and evaluates their dependence on CAM.

## 2 CAM functioning

CAM is a water-conserving mode of photosynthesis that enables CO_2_ concentration around ribulose-1,5-bisphosphate carboxylase/oxygenase (Rubisco) despite closed stomata and is characterized by distinct diurnal phases in carbon metabolism. At night (phase I), atmospheric CO_2_ is fixed by phosphorylation-activated phosphoenolpyruvate carboxylase (PEPC), forming oxaloacetate that is subsequently reduced to malate and accumulated in the vacuoles. In the morning, there is a transition of the primary CO_2_-fixing enzyme from PEPC to Rubisco (phase II). During the daytime, stored malate is decarboxylated, and the released CO_2_ is then fixed by Rubisco and reduced in the Calvin-Benson-Bassham cycle while stomata remain closed (phase III) (**Figure 1D**). When environmental conditions are favourable or the malate supply is exhausted, stomata may open in the late afternoon (phase IV), allowing direct uptake of atmospheric CO_2_, which supplements their total carbon intake (Osmond, 1978; Holtum et al., 2005). This diurnal cycle is reflected in leaf malate concentration, the activity of PEPC and its activating enzyme PEPC kinase, as well as in the cellular ATP/ADP ratio (Winter, 1982; Taybi et al., 2000; Niewiadomska et al., 2004). The molecular mechanisms underlying NaCl-induced C_3_-CAM shift in *M*. *crystallinum* remain obscure. However, recent analyses have highlighted the importance of protein phosphorylation, abscisic acid signalling, inositol metabolism, and vacuolar H^+^-ATPase activity (Guan et al., 2024; Toyokura et al., 2025; Tan et al., 2025).

CAM depends on the PEPC activity and requires a night-time supply of three-carbon skeletons for cytosolic phosphoenolpyruvate (PEP) synthesis. In *M. crystallinum*, this supply is secured by increased degradation of starch (**Figure 1D**) (Paul et al., 1993; Borland et al., 2009). In addition to providing carbon skeletons, starch breakdown followed by glycolytic conversion of glucose-6-phosphate to PEP in the cytosol generates ATP, which may support vacuolar H^+^-ATPase activity and thereby contribute to the energization of nocturnal malate accumulation in the vacuole (Neuhaus and Schulte, 1996). The importance of starch degradation for CAM was confirmed in starch-deficient mutant plants lacking plastidic phosphoglucomutase, which fail to induce CAM under saline conditions (Cushman et al., 2008; Haider et al., 2012). Although this mutant is characterised by slower growth and reduced seed production under salinity, these plants survive and reproduce (Cushman et al., 2008), indicating that in the ice plant, basic salinity resistance is present independently of CAM induction.

## 3 PET performance

The C_3_-CAM shift is an adaptation to habitats subjected to seasonal changes in water availability and seasonal flooding with seawater. The rapid juvenile growth characteristic of *M. crystallinum* appears to represent a mechanism that establishes a sufficient biomass of C_3_ photosynthetic tissue capable of supporting the increased demand for reserve carbohydrates following CAM induction (Winter and Ziegler, 1992; Adams et al., 1998). Leaves developing in juvenile plants exhibit efficient PET, leading to the accumulation of transitory starch in chloroplasts. However, growth under non-saline conditions exerts a negative effect on electron transport in mature leaves, as indicated by plastoquinol oxidase (PTOX) activity, and by a decline in the whole chain electron transport (from water to ferricyanide) accompanied by enhanced PGR5-dependent cyclic electron transport (CET) (Niewiadomska and Pilarska, 2021). In addition, plants grown under non-saline conditions exhibit signs of senescence, as evidenced by the accumulation of the senescence-associated protein SAG12. In contrast, in salt-acclimated plants with induced CAM, the decline of linear electron transport (LET) is prevented, and chloroplastic alternative electron sinks observed in C_3_ plants are not stimulated (Niewiadomska and Pilarska, 2021). In CAM plants, the generation of PEP from pyruvate in chloroplasts during the day requires ATP, consumed by pyruvate orthophosphate dikinase activity (Kondo et al., 1998), and a possible mechanism for producing this extra energy is CET around PSI. Therefore, the lack of CET enhancement is rather surprising and indicates that there is another source of this extra ATP in ice plant chloroplasts. In some halophytic species lacking CAM induction, PTOX activity under salinity is considered a tolerance mechanism (Stepien and Johnson, 2009; Uzilday et al., 2015). Thus, the absence of this alternative electron sink in *M*. *crystallinum* seems unique and could be linked to C_3_-CAM transition. This effect might be caused by a high turnover of NADPH, preventing over-reduction of electron carriers, as it is well accepted that plants activate the alternative electron sinks to avoid electron accumulation in PET chain (Demircan et al., 2024). However, inefficient PSI oxidation by far-red light (Niewiadomska and Pilarska, 2021) indicates over-reduction of downstream electron sinks.

The prevention of PSII photoinhibition under salinity appears to be a common feature of halophytes (Bose et al., 2017). In *M*. *crystallinum*, acclimation to salinity results in increased activity of not only PSII, but also PSI, as shown by independent measurements of PSI- and PSII-mediated electron transfer in isolated thylakoid membranes using artificial electron acceptors (Niewiadomska and Pilarska, 2021; Pilarska et al., 2023). Immunoblotting revealed no quantitative changes in the protein levels of PSII (D1) or PSI (PsaB) (Pilarska et al., 2025). However, this method may not be sensitive enough to detect minor changes in the amount of these photosystems. It is hypothesised that the higher PSII activity could be a result of its more efficient repair, as indicated by stronger D1 phosphorylation (Pilarska et al., 2025).

## 4 Changes in PQ pool redox state

In a well-accepted scenario applicable to unstressed plants, the redox state of the plastoquinone (PQ) pool changes day-night in a manner that it is partly reduced during the day under functioning PET and mostly oxidized at night when electron transport is suppressed (Havaux, 2020). Induction of PTOX in ageing leaves under non-saline conditions is expected to contribute to an oxidation of the photochemically active PQ pool. Indeed, HPLC analyses revealed a highly oxidized PQ pool during the day (Pilarska et al., 2023). In contrast, the degree of PQ pool reduction increases significantly in plants acclimated to salinity. Since the involvement of CET has been excluded (Niewiadomska and Pilarska, 2021), a possible explanation for this phenomenon is suppressed photosynthetic control, which is a way of PET intensity regulation by feedback control by NADPH, ΔpH and ATP synthase conductivity on the plastoquinol oxidation step at cytochrome b_6_f (Degen and Johnson, 2024). Support for this interpretation comes from lower non-photochemical quenching (NPQ) (Niewiadomska and Pilarska, 2021) and lower steady-state P700 oxidation (Y(ND)) (Niewiadomska et al., 2011).

Despite the highly reduced PQ pool during the day, PET performance in salt-acclimated, CAM-performing *M*. *crystallinum* plants does not appear to be impaired. This is probably due to an enlargement of the photochemically active PQ pool at the expense of photochemically nonactive fraction, as shown by HPLC analyses (Pilarska et al., 2023). Functional assays based on the PQ reduction in leaf discs confirmed that in these plants, the larger pool of PQ can be reduced by PSII. This finding correlates with facilitated electron flow through PSII, as indicated by non-invasive chlorophyll *a* fluorescence measurements revealing an increased pool of open PSII reaction centres (RCs) (Pilarska et al., 2023). Such a highly increased capacity of the PQ pool for electrons from PSII in salt-acclimated plants can therefore be regarded as a mechanism supporting electron transport under salinity. As demonstrated in other species, changes in the ratio between photochemically active and nonactive PQ take place in response to light intensity (Szymańska and Kruk, 2010; Suslichenko and Tikhonov, 2019), thus this effect is likely CAM-independent.

At night, when electron transport is suppressed, the chloroplast redox state is under metabolic control, and the photochemically active PQ pool is mostly oxidized (Havaux, 2020). However, before dawn in salt-acclimated ice plant, the PQ pool is even in a more reduced state than during the day (Pilarska et al., 2023). In *Arabidopsis thaliana*, nocturnal reduction of the PQ pool has been attributed, at least in part, to the activity of the NADPH dehydrogenase-like complex, which utilizes stromal reductants (Nellaepalli et al., 2012). In salt-acclimated ice plant, this phenomenon may be linked to altered carbon metabolism associated with intensive nocturnal starch breakdown characteristic of CAM plants (Haider et al., 2012).

## 5 The capacity of light harvesting

The redox state of the PQ pool influences many processes, including the phosphorylation of photosynthetic proteins (Havaux, 2020). As shown by immunoblotting, in salt-acclimated *M*. *crystallinum*, the phosphorylation of PSII light-harvesting complex (LHC) proteins, LHCB1 and LHCB2, is higher than in C_3_ controls both during the day and at night (Pilarska et al., 2025), which is consistent with a greater reduction of the PQ pool. Excitation of PSII and PSI should be adjusted to optimise photochemistry. This can be achieved through state transitions, which involve phosphorylation-controlled changes in the relative size of PSII and PSI antennae (Longoni and Goldschmidt-Clermont, 2021; Shang et al., 2023). Typically, in darkness, LHCII are mostly dephosphorylated and their mobile fraction is bound to PSII (State I), while in the light, part of phosphorylated LHCII associates with PSI (State II). In salt-acclimated ice plant, functional analyses based on changes in chlorophyll fluorescence in response to different light quality revealed that the effectiveness of state transitions is impaired (Pilarska et al., 2025). Additionally, low-temperature chlorophyll emission spectra showed that day-night variations in the relative size of the PSI antenna are less prominent, and at night a part of mobile LHCII remains associated with PSI, indicating a permanent State II (Pilarska et al., 2025). In CAM plants, the values of the fast chlorophyll *a* fluorescence induction kinetics parameters describing energy flow through PSII were lower than in C_3_ plants, confirming a decrease in the relative size of PSII antenna (Pilarska et al., 2025). Although a reduction in PSII light harvesting antenna size is considered a universal acclimation mechanism in higher plants under stress (Borisova-Mubarakshina et al., 2020), it seems that it is not a typical response of halophytes to salinity (Niewiadomska and Wiciarz, 2015). Nevertheless, in some halophytic species, PSII antenna size reduction has been achieved through quantitative changes rather than through functional adjustments (M’rah et al., 2006; Rabhi et al., 2010).

## 6 Conclusions and future perspectives

The mechanisms involved in acclimation of photosynthetic electron transport to salinity in the facultative CAM species *M. crystallinum* are summarized in **Table 1**. It can be concluded that the redox dynamics of the PQ pool play a central role in PET acclimation. Importantly, the influence of the PQ redox state is likely to extend beyond electron transport, as it also affects the expression of key genes encoding ROS scavengers and activity of cytoplasmic NADP-dependent malic enzyme (Ślesak et al., 2002).

**Table 1:** Summary of the processes discussed in this review that contribute to PET acclimation to salinity in *Mesembryanthemum crystallinum*, in comparison with C_3_ plants.

Process/Feature	Observed state/Effect	Proposed role/Significance
overall PET performance	supressed decline of LET; no CET enhancement, PTOX not active (Niewiadomska and Pilarska, 2021)	ensures unchanged electron flow through LET
PQ pool	PQ pool in a reduced state (day & night); increased size of the photo-reducible PQ pool (Pilarska et al., 2023)	maintains high electron flow capacity; source of redox signalling
changes in PSII and PSI	increased PSII (Pilarska et al., 2023) and PSI activity (Niewiadomska and Pilarska, 2021); enlarged pool of open PSII RCs (Pilarska et al., 2023); increased D1 phosphorylation (day & night) (Pilarska et al., 2025)	prevents photoinhibition
LHCII phosphorylation, state transitions	increased LHCII phosphorylation (day & night); permanent state II; decrease in the relative size of PSII antenna (Pilarska et al., 2025)	limits excess excitation energy

It is reasonable to speculate that mechanisms enabling *M*. *crystallinum* to adapt the light reactions of photosynthesis to salinity reflect both CAM-dependent and CAM-independent effects. However, a clear distinction between these effects is not yet possible. It therefore remains an open question whether the highly reduced state of the PQ pool at night should be considered a CAM-dependent phenomenon. Starch degradation and CAM induction are closely linked, as salt exposure simultaneously increases the transcript abundance of genes required for nocturnal carboxylation, as well as for starch and sucrose degradation in both wild-type plants and starch-deficient mutants impaired in CAM induction (Taybi et al., 2017). It is anticipated that the development of additional CAM-deficient mutants, for example through suppression of the CAM-specific form of PEPC, will facilitate a separation of CAM-dependent and CAM-independent effects of salinity in this species.

A close relationship exists between the light phase of photosynthesis and the enzymatic reactions in chloroplasts that fulfil the changing demands for ATP and NADPH. In this respect, it is known that the energy requirement for CO_2_ fixation is higher in CAM plants than in C_3_ plants (Winter and Smith, 1996). Therefore, given the absence of CET stimulation in CAM-performing *M*. *crystallinum*, it seems important to conduct detailed studies on ATP and NADPH formation during PET, with particular emphasis on the mechanisms regulating chloroplast ATP synthase activity.
